# Genistein anticancer efficacy during induced oral squamous cell carcinoma: an experimental study

**DOI:** 10.1186/s43046-022-00140-5

**Published:** 2022-09-05

**Authors:** Ahmed M. Hussein, Abdelraheim H. Attaai, Asmaa M. Zahran

**Affiliations:** 1 Department of Oral and Maxillofacial Pathology, Faculty of Dentistry, Assiut University, Assiut, Egypt; 2 Department of Anatomy and Embryology, Faculty of Veterinary Medicine, Assiut University, Assiut, 71526 Egypt; 3 Department of Clinical Pathology, South Egypt Cancer Institute, Assiut University, Assiut, Egypt

**Keywords:** Genistein, DNA ploidy, Flow cytometry, S-phase fraction

## Abstract

**Background:**

About 7 million people die from various types of cancer every year representing nearly 12.5% of deaths worldwide. This fact raises the demand to develop new, effective anticancer, onco-suppressive, and chemoprotective agents for the future fighting of cancers. Genistein exhibits pleiotropic functions in cancer, metabolism, and inflammation. It functions as an antineoplastic agent through its effect on the cell cycle, apoptotic processes, angiogenesis, invasion, and metastasis.

**Aim of the study:**

The current study aimed to study the genistein onco-suppressive effects during 7,12-dimethylbenz[a]anthracene (DMBA)-induced oral carcinogenesis in hamsters’ buccal pouch utilizing flow cytometry analysis (FMA), as a fast-diagnosing tool, in addition to the histopathology.

**Material and methods:**

The buccal mucosa of adult male Syrian hamsters was painted with paraffin oil only (group 1), DMBA mixed in mineral oil (group 2), or orally administrated genistein along with painting DMBA (group 2B). The buccal mucosa was utilized for flow cytometric analysis and histopathological examination.

**Results:**

Grossly, DMBA-induced carcinogenesis started at the 9th week. Progressive signs appeared in the following weeks reaching to large ulcerative oral masses and exophytic nodules at the 21st week. Histologically, invasive well-differentiated oral squamous cell carcinoma (OSCC) appeared in the underlying tissues from the 12th week, showing malignant criteria. Genistein had delayed clinicopathological change, which started 6 weeks later, than the DMBA-painted hamsters, as mild epithelial dysplastic changes. This became moderate during the last 6 weeks, without dysplastic changes. Flow cytometry revealed that DMBA led to considerable variation in DNA proliferation activity, aneuploid DNA pattern, in 47.22% of hamsters and significantly raised the S-phase fragment (SPF) values, which drastically reduced after genistein treatment.

**Conclusion:**

Taken together, genistein could be employed as an onco-suppressive agent for carcinogenesis. Moreover, FMA could be used as an aiding fast tool for diagnosis of cancer.

## Background

The rate of cancer incidence, according to IARC-WHO estimates, was expected to increase at an alarming rate: from 10 million new cases globally in 2000 to 15 million in 2020 [1]. Cancer arises in a multistage process that generally originated from a precancerous lesion to a malignant tumor [2]. The oral cavity is the gateway to the alimentary and respiratory tracts, so it is subjected to numerous carcinogenic agents. The oral squamous cell carcinoma is characterized by a high level of morbidity and mortality. Despite the significant advances in therapeutic strategies, the low survival rate for oral cancer patients remains at approximately half of the cases [3]. A chemopreventive path might be an effective alternative to the old current therapies [4]. Fresh vegetables and fruits are excellent sources of cancer-preventive substances. They intervene with the carcinogenesis process leading to slow down, arrest, or reverse it. The natural substances separately or in combination to therapy have been emerging as a promising approach to diminish the malignancy hazard [5].

Genistein constitutes the major component of isoflavone, which presents in high-soy diets. Owing to its chemopreventive and therapeutic effects, genistein has received much attention. It targets numerous cellular signal transduction pathways associated with cell cycle regulation and apoptosis. In addition, genistein has been suggested to have antiangiogenic and antioxidant activities [6].

DNA content is viewed as a marker of the cell position within the cell cycle. The normal nondividing cells are diploid cells, in a resting state, G0 phase. After signals for proliferation, they enter the G1 phase, where they keep their ploidy by retaining two complete sets of chromosomes (2 N). As the cells enter the S phase, DNA replication starts, and cells contain varying amounts of DNA. The DNA content reaches a tetraploid state (4 N), which become twice the DNA content of the diploid state. Tetraploid cells in the G2 phase start preparing for division and enter the M phase when the cells divide into two identical diploid (2 N) daughter cells. The daughter cells proceed on to another division cycle or enter the resting stage G0 [9]. Nuclear DNA content of a cell can be quantitatively estimated at high speed by flow cytometry (FCM), which measures and analyzes various physical characteristics of single cell, while cells flow in a fluid stream through a beam of light or laser [10]. The cell ploidy status, DNA index (DI), and the percent of cells in synthesis phase fraction (SPF) are the major parameters used in the FCM for identification and classification of tumors cell activity [11].

The hamster buccal pouch (HBP) carcinogenesis model is a well-characterized tumor model representing a paradigm for oral carcinogenesis. The HBP is easily accessible for tumor induction and application of test agents without the need for anesthesia. The pouch can be readily subjected to gross examination and follow-up of lesions. Therefore, it is ideal for analyzing the stepwise advancement of oral cancer and the effect of chemo-intervention. The development of oral cancer is a multistep process requiring initiation, promotion, and progression [12].

Application of DMBA to the buccal pouch of the Syrian hamster produces squamous cell carcinoma and premalignant lesions that are histologically similar to that in humans [13]. After 6 weeks of DMBA application, mucosal lesions of the cheek pouches are maximally at the premalignant stage, which are comparable to human cases with oral leukoplakia or in heavy smokers. This post-initiation stage could be a good time to test the primary preventive effect of chemopreventive agents [14]. The aim of the current study was to investigate the chemoprevention impacts of genistein on oral carcinogenesis process in HBP at the post-initiation stage, as well as its effect during the carcinogenesis process.

## Material and methods

### Animals grouping

A total of 95 male Syrian golden hamsters were purchased from Theodor Bilharz Research Institute, Cairo, Egypt. They were clinically healthy, 8 weeks old, and weighing about 100 g. The animals were housed in show polypropylene cages (5 per cage) in a room with controlled temperature and humidity under 12-h light/dark cycles. All the experiments were conducted at the Experimental Animal Unite, Oral and Maxillofacial Pathology Research Institute. All animal proceedings were conducted following the National Institute of Health Guide for the Care and Use of Laboratory Animals [15]. Animals were provided with sterilized soy-free diet, comprising 16% protein and tap water ad libitum.

The full 25 weeks of the study were planned as the following: a week of acclimatization, after which a single hamster was sacrificed, after euthanized by ether inhalation, and used for histological and FCM examination of normal HBP mucosa. The remaining 94 animals were randomly divided into 2 main groups: group 1 (as the control group, *n* = 16), the right buccal pouches were painted, 3 times per week, with a heavy mineral oil only, and group 2 (*n* = 78), where the right HBP were painted 3 times a week with 0.5% DMBA (Sigma, USA) dissolved in mineral oil, using number 4 sable-hair brush [16]. During the carcinogenesis process, the animals were examined regularly for clinical evaluation, and every 3 weeks, three animals were victimized. After 6 weeks of painting DMBA, the remaining 72 hamsters were randomly divided into 2 subgroups as the following: group 2A (*n* = 36), where the HBP of animals were painted with DMBA only: group 2B (*n* = 36), where genistein (Sigma-Aldrich, USA) was orally administrated as a chemoprotective agent, concurrently with DMBA painting. Genistein was given as a suspension in distilled water by gavage, 20 mg/kg animal body weight/day [17]. For visualization of the carcinogenesis processes, the animals were examined frequently for clinical evaluation. The skin of the right buccal pouch mucosa was retracted, and the medial wall was examined carefully for any gross pathological changes. For histological and flow cytometric analysis, every 3 weeks, 2 animals were sacrificed from group 1 and 6 animals from each group 2A and 2B.

### Flow cytometry analysis

The buccal mucosa samples of the painted side from all animals were collected for FCM and histopathological examination. Three sections from each selected tumor tissue, of nearly 30 μm thickness, were placed into labeled glass culture tubes and transferred, on ice, to FCM Unit, Clinical Pathology Department, South Egypt Cancer Institute, Assiut University. Samples were analyzed using a FACS Calibur Flow Cytometer (Becton Dickinson Biosciences, San Jose, California, USA). The DNA was stained by the CycleTEST™ PLUS DNA Reagent Kit (BD Biosiences). The cell cycle phases and the DNA indices of the nuclear clones were calculated using the ModFit Software Package. The DNA diploid number of normal HBP was used as a reference for the identification of aneuploid DNA clones.

### Histopathological analysis

The remaining of the HBP, from each animal, was fixed in 4% PFA and processed for paraffin embedding procedure. Every tenth serial sections from each sample were stained with hematoxylin and eosin (H&E) for histopathological analysis. Basal cell hyperplasia, papillomas, dysplasia, in situ carcinoma, and squamous cell carcinoma were determined. Increased number of basal cells was considered as a *hyperplasia* of oral epithelium. *Papilloma* was identified by proliferation of stratified squamous epithelium. Irregular epithelial stratification, alteration of nuclear-cytoplasmic ratio, increased numbers of mitotic activity, and loss of basal cells polarity were categorized to be *epithelial dysplasia*. Top to bottom epithelium dysplasia indicating *carcinoma* in situ (CIS). Moreover, *carcinoma* was identified by epithelium invasion of the underlying connective tissues.

### Data management and statistical analysis

Tumors with a single G0/G1 peak with DI of 0.95 to 1.05 to the reference sample were classified as DNA diploid. If two discrete G0/G1 peaks were present, with an abnormal G0/G1 peak containing a minimum of 15% of the total events and having a corresponding G2/M peak, then the tumors were considered DNA aneuploid [18]. The DI was recorded by the calculation program for DNA analysis system. Therefore, samples were considered hypodiplod if their DI was less than 0.95 or hyperdiploid if their DI was more than 1.05. The SPF is the fraction of the total cell populations that are present in the S phase of the cell cycle and is usually expressed as a percentage. The cutoff for the SPF was set as the mean ± 2 standard deviation (SD) and considered as either being low or high.

Data were statistically described in terms of mean ± SD, median and range, or frequencies and percentages when appropriate. Comparison of FCM variables between the study groups was done using Mann–Whitney *U*-test and Kruskal–Wallis test. For comparing categorical data, chi-square (± 2) test was performed. *P*-value less than 0.05 was considered statistically significant. All statistical calculations were done using computer programs (Statistical Package for the Social Science; SPSS Inc., Chicago, IL, USA) version 15 for Microsoft Windows.

## Results

### Clinical and histopathologic evaluation

The current study was performed on adult gold Syrian hamsters. Every 3 weeks of the study, the animals were clinically evaluated, and representative number from the groups was sacrificed for FCM and histological examination.

### Control group (group 1)

**Fig. 1:**
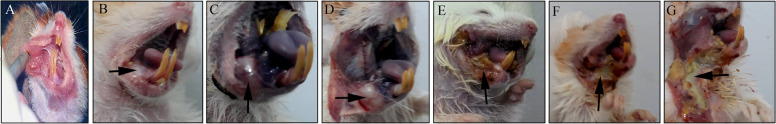
A plate of clinical pictures during treatment with DMBA and genistein. The HBP showing **A** no clinical changes, group 1; **B** areas of white patches at 4 weeks, group 2; **C** moderate exophytic nodule at 15 weeks, group 2A; **D** exophytic mass at 18 weeks, group 2A; **E** ulcerative lesion at 21 weeks, group 2A; **F** endophytic ulcers with necrotic floor at 24 weeks, group 2A; **G** massive areas of necrosis and extensive tissue destruction at 24 weeks, group 2B

The HBP of 16 animals were painted with paraffin oil only. Neither clinical nor histological evidence of pathological changes was noticed during the whole experimental period (Figs. 1A–A). Hyperkeratosis, without any dysplastic changes, was merely observed in this group.

### Validation of DMBA-induced carcinogenesis model

#### The first 6 weeks of DMBA painting (group 2)

**Fig. 2:**
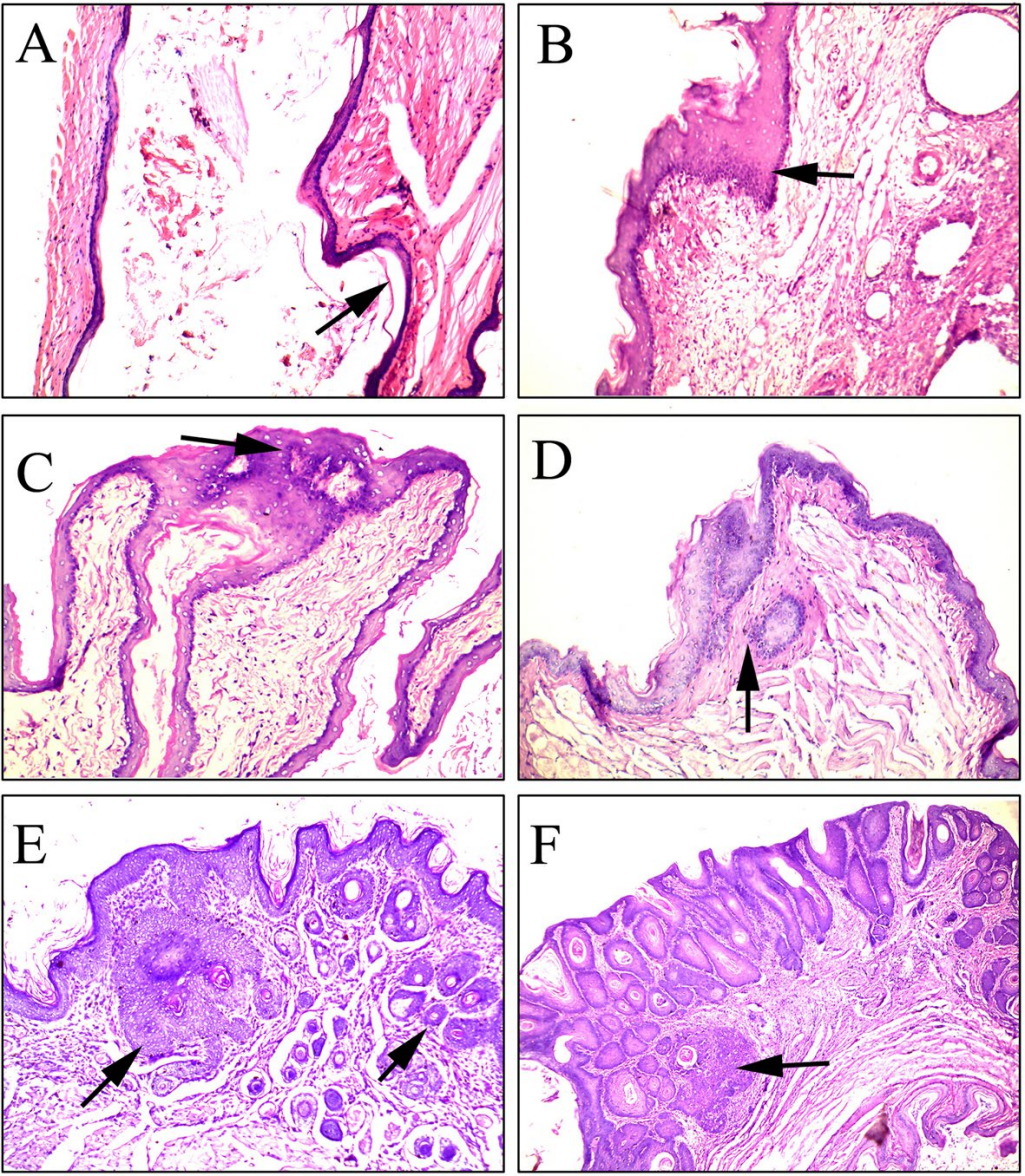
A photomicrograph showing induced carcinogenesis during treatment with DMBA. **A** Normal epithelium lining of hamster buccal pouch, group 1. **B** Focal thickening without any cellular atypia, group 2A. **C** Mild dysplasia, group 2A. **D** Microinvasion of the malignant epithelial cells into underlying tissue, group 2A. **E** Invasive well-differentiated OSCC, group 2A. **F** Invasive well- to moderate-differentiated OSCC, group 2A. All pictures were stained by H&E and taken at × 100 except 2A and 2F at 40 ×

Grossly, small areas of white patches (Fig. 1B) were observed in some hamsters. Histologically, the lining epithelium of the examined HBPs revealed areas of focal thickening without any cellular atypia (Fig. 2B and Table 1). After that, the remaining 72 animals are divided into 2 subgroups. At 9, 12, 15, 18, 21, and 24 weeks of the study, six animals from each subgroup were examined for any pathological changes as the following:

**Table 1:** The summary of the histopathological finding and flow cytometry (FCM) analysis of hamsters’ buccal pouch. The tumorigenesis has been induced using DMBA. Genistein has been administrated orally to group 2B as a possible chemo-suppressor for tumorigenesis. *SPF*, S-phase fragment; *w*, week; *n*, number of animals

W	*n*	Group 2						
3 W	3	No clinical or histopathological changes (FCM analysis: diploid, low SPF)						
6 W	3	White patch with epithelial hyperplasia and no cellular atypia (diploid, low SPF)						
Group 2A (DMBA)					Group 2B (DMBA + genistein)			
W	*n*	Histopathological finding	FCM analysis		*n*	Histopathological finding	FCM analysis	
			Diploid/aneuploid	SPF L/H			Diploid/aneuploid	SPF L/H
9 w	2	Epith. hyperplasia	2/0	2/0	6	Epith. hyperplasia with no cellular atypia	6/0	6/0
	4	Mild dysplasia and papilloma	4/0	2/2				
12 w	1	Moderate dysplasia	1/0	0/1	5	Epith. hyperplasia	5/0	5/0
	3	Carcinoma in situ	2/1	2/1	1	Erythema, mild dysplasia	1/0	0/1
	2	Early invasive carcinoma	2/0	1/1				
15 w	2	Early invasive carcinoma	1/1	1/1	3	Epith. hyperplasia	3/0	3/0
	4	Invasive, well-differentiated OSCC	3/1	1/3	3	Mild dysplasia endo- and exophytic epithelial growth	3/0	2/1
18 W	4	Invasive, well-differentiated OSCC	2/2	1/3	2	Epith. hyperplasia	2/0	2/0
	2	Moderate-differentiated OSCC	1/1	1/1	3	Mild epithelial dysplasia	1/2	1/2
					1	Early invasive carcinoma	0/1	0/1
21 W	1	Invasive, well-differentiated OSCC	0/1	0/1	3	Moderate dysplasia	3/0	2/1
	5	Ulcerative oral mass, moderate-differentiated OSCC	1/4	2/3	2	Early invasive carcinoma	1/1	1/1
					1	Invasive, well-differentiated OSCC	0/1	0/1
24 W	2	Moderate-differentiated OSCC	0/2	0/2	3	Necrotic areas without any dysplasia	3/0	3/0
	4	Poor differentiated OSCC, malignant criteria	0/4	0/4	2	Invasive, well-differentiated OSCC	1/1	0/2
					1	Moderate OSCC	0/1	0/1
Total	36	29 carcinoma	19/17	13/23	36	10 carcinoma	29/7	26/11
%		80.56% carcinoma	47.22% aneuploid	63.89% H SPF		27.78 carcinoma	19.44% aneuploid	30.56% H SPF

#### The second 6 weeks (7–12) of DMBA painting (group 2A)

At the 9th week, non-scrapable raised red to white lesions that showed mild dysplasia, and papilloma started to appear at the HBPs (Fig. 2C). At the 12th week, ulcerative lesions and hyperemic HBPs were observed in most cases. Carcinoma in situ was noted in half of the group. Two animals demonstrated areas of microinvasion of the malignant epithelial cells into the underlying tissues (Fig. 2D and Table 1).

#### The third 6 weeks (13–18 of DMBA painting (group 2A)

At the 15th week, the pouches in most animals developed moderate exophytic nodules (Fig. 1C). Examined HBPs developed invasive, well-differentiated OSCC in 4 animals; however, only early invasion appeared in the remaining 2 animals (Fig. 2E and Table 1). By the end of the 18th week, well-developed oral tumors were observed, either as exophytic masses or endophytic ulcers (Fig. 1D). The lining epithelium showed features of well- to moderate-differentiated OSCC (Fig. 2F and Table 1).

#### The final 6 weeks (19–24) of DMBA painting (group 2A)

**Fig. 3:**
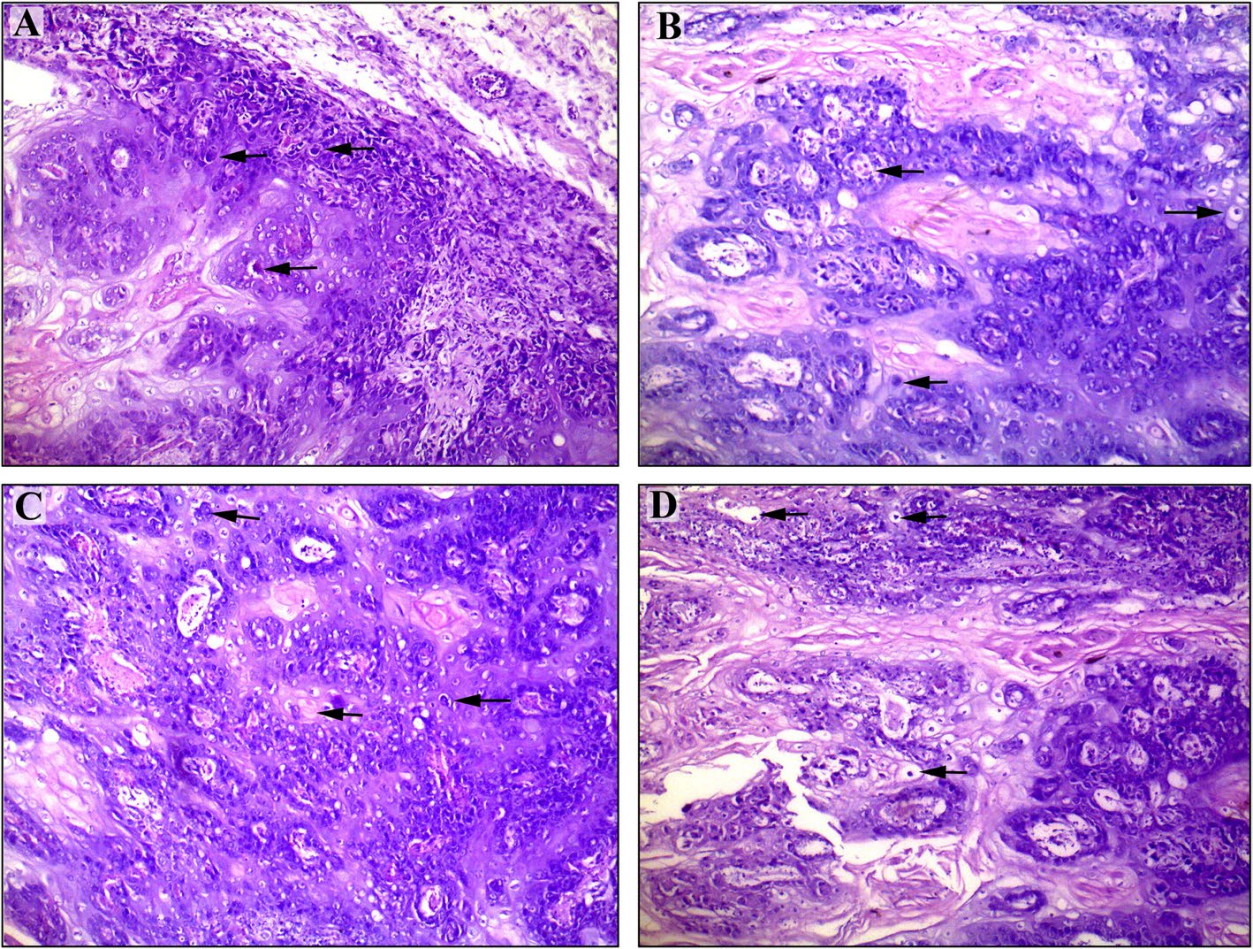
A photomicrograph showing different fields in poorly differentiated OSCC with evident malignant criteria demonstrating **A** hyperchromatism, **B** pleomorphism, **C** loss of polarity, and **D** abnormal nuclear cytoplasmic ratio and abnormal mitosis, 24 weeks after exposure to DMBA, group 2A (H&E × 100)

At the 21st week, large ulcerative oral masses developed in the HBPs (Fig. 1E). The histological examination revealed moderate-differentiated OSCC in 5 animals. The remaining animals developed huge fungal oral masses at the 24th week (Fig. 1F). The invading epithelial cells showed malignant criteria such as pleomorphism, hyperchromatism, loss of cellular adhesion, and altered nuclear cytoplasmic ratio. Abnormal mitotic figures were observed as a characteristic feature in the poorly differentiated type (Fig. 3 and Table 1).

### Effects of genistein on the DMBA-induced tumors (group 2B)

#### During the first 12 weeks

The animals were orally administrated genistein, 6 weeks after application of DMBA, as a chemoprotective agent. No clinicopathological changes appeared in the HBPs of most of the animals. Variable areas of erythema were developed, merely in few hamsters (Table 1).

#### During the second 6 weeks

After 15 weeks, endophytic and exophytic epithelial growth with hyperkeratosis were detected. At the 18th week, few animals developed exophytic nodules in the buccal pouches. Mild epithelial dysplastic changes were demonstrated in 3 hamsters, focal area of early invasive carcinoma in one hamster; however, no pathological changes were seen in the remaining 2 hamsters (Table 1).

#### During the final 6 weeks

**Fig. 4:**
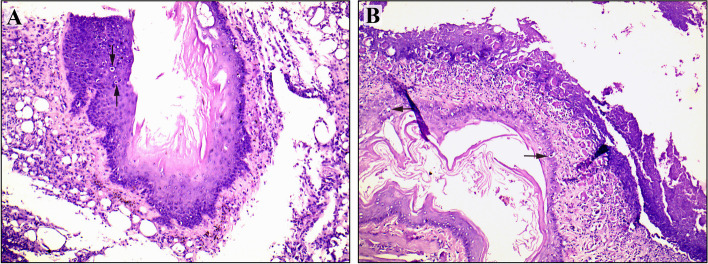
A photomicrograph showing apoptotic malignant epithelium cells (arrows) with condensed nucleus and clearance of cytoplasm at 21 weeks of DMBA and genistein administration, group 2B (H&E × 100)

At the 21st weeks, the HBPs of few animals exhibited several exophytic nodules. White patches were observed grossly in nearly 50% of the hamsters. Moderate to severe epithelial dysplastic changes appeared in half of the examined buccal pouches. Some areas revealed early invasive OSCC. In addition, apoptotic malignant epithelial cells, with condensed nuclei and cleared cytoplasm, were detected (Fig. 4 and Table 1). At the end of the study (at the 24th week), the HBPs tissue of 3 animals revealed well and moderate-differentiated OSCC. In the remaining 3 hamsters, massive areas of necrosis (Fig. 1G) with mild hyperemia of the underlying tissue were noted, but without any dysplastic changes (Table 1).

The current results indicate that the cancer incidence had a range of variation between the study groups. The difference in cancer incidence was highly statistically significant (*p* < 0.0001) when comparing between group 1 and group 2A. In addition, when comparing between group 1 and group 2B. Moreover, the difference in cancer incidence was highly statistically significant (*p* < 0.0001) when comparing between group 2A and group 2B.

### Flow cytometric analysis

A total number of 95 HBPs, from 95 used hamsters, were analyzed by DNA-FCM.

#### The DNA ploidy state

**Fig. 5:**
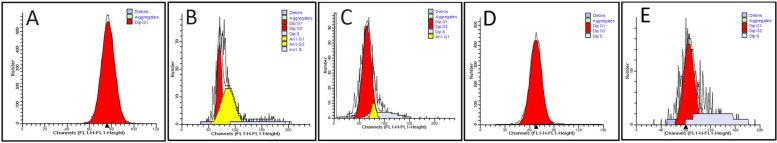
Flow cytometry analysis of the buccal mucosa DNA. DNA frequency histogram of **A** diploid peak obtained from the normal oral HBP mucosa, showing single G0/G1 peak and no SPF cells. **B** Aneuploid malignant tumors in group 2A showing hyperdiploid (*DI* = 1.22) and high SPF cells (12.82%). **C** Aneuploid malignant tumors in group 2B showing hyperdiploid (*DI* = 1.20) and high SPF cells (15.83%). **D** Animals in group 1, showing low SPF (1.25%) and diploid peak. **E** Animals in group 2A, showing high SPF (33.26%) and diploid peak

The standard single diploid peak, obtained from the normal oral HBPs mucosa, was considered the reference peak representing G0/G1 cells (2 N) (Fig. 5A). All animals in group 1, which were painted with paraffin oil only, were diploid with few cells in the S phase or at G2/M peak. After induction of carcinogenesis with DMBA, 17 out of 36 animals (47.22%) in group 2A showed aneuploid DNA pattern with considerable variation in DNA content (Fig. 5B and Table 1). On the other hand, this number decreased after treatment with genistein, where only 7 hamsters (19.44%) of group 2B developed aneuploid DNA pattern (Fig. 5C and Table 1). The aneuploid tumors were either hyperdiploid or hypodiploid. In hyperdiploid cases, DI ranged from 1.06 to 1.53 with a mean of 1.28 (12 in DMBA group versus 3 in genistein-treated group). Whereas in hypodiploid cases, DI ranged from 0.76 to 0.92 with a mean of 0.87 (5 in DMBA group versus 4 in genistein-treated group). The difference in diploid and aneuploid DNA pattern (the ploidy state) between group 1 and group 2A or group 2B and between group 2A and group 2B and the hyperdiploid aneuploidy and the tumorigenesis was statistically highly significant (*p* = 0.001). There is no significant difference in the number of hypodiploid aneuploidy between group 2A and group 2B (*p* = 0.572).

#### The S-phase fragment values (SPF)

The calculated SPF values for the control group were very low, ranged between 0 and 2.17%, with a mean of 1.65% (Fig. 5D and Table 1). After induction of carcinogenesis with DMBA, in group 2A, SPF values raised significantly (*p* = 0.001) to reach up to 47.53% with a mean of 22.37% in 63.89% of animals (23/36 cases). Meanwhile, genistein treatment significantly (*p* = 0.001) reduced the number of cases having high SPF, where 69.44% (25/36) of cases had low SPF value: 6.82% and 23.71% with a mean of 11.65% (Fig. 5E and Table 1).

## Discussion

Oral carcinoma is one of the most common cancers of the head and neck, with a poor prognosis. A better understanding of the molecular mechanisms underlying the development and progression of OSCC will help identify novel targets for pharmacological intervention and chemoprevention of this disease [19].

Genistein is being studied by many researchers as an onco-protective agent for many tumors of different systems. The oral administration of genistein inhibited the formation of lung tumors and decreased their volume [20]. Genistein may also exert beneficial antitumor effects to inhibit the development and progression of human prostate cancer and gallbladder [21, 22]. Moreover, genistein exerted a chemopreventive activity against the intestinal tumorigenesis and the colon cancer cells [23]. Furthermore, genistein has a strong therapeutic potential in animal model of hepatic carcinomas [24]. Therefore, the present study aimed to determine using genistein as a potential onco-protective agent for OSCC and to evaluate the diagnostic values of the flow cytometry for OSCC.

Profound studies showed that genistein has a role in the induction of apoptosis, inhibition of the cell proliferation, and modification of the cell cycle progression. Further support can also be derived from the in vitro observation that genistein induced cell growth inhibition and apoptosis in head and neck SCC cell lines [25]. These observations are comparable to the results of the present work, where apoptosis was detected in some carcinomatous tissues after genistein administration. In agreement with the results of the present study, Polivkova and colleagues reported the antimutagenic and anti-genotoxic effects of genistein against the mutagens and carcinogens materials [26]. Moreover, the oral administration of chemoprotective agents as genistein reversed the frequency of the carcinogenesis process [27–29]. Furthermore, Hussein and colleagues concluded that genistein provides a chemoprotective role during the process of oral carcinogenesis [30], and it has been suggested as chemotherapeutic agent for oral carcinomas [32].

In contrast, Yang and colleagues did not find any chemopreventive effect of genistein on DMBA-induced oral carcinogenesis [31]. This might be due to the very low dosage of genistein, relevant to the real-life consumption pattern (0.3–1 mg/kg). Moreover, Myoung group did not find any inhibitory effect for genistein on the transplanted human OSCC [33], which could be attributed to their tumor models which usually contained newly formed immature blood vessels that interfered with the effects of most antiangiogenic drugs. The variation in the genistein concentrations exerts different effects on the carcinogenesis process. Moreover, the timing of genistein administration may also affect the metabolism, bioavailability, and its biological action. For instance, the administrated dose of genistein after a long period of exposure to DMBA may not be enough to maximize the chemoprotective effect of genistein. This could be explained that it takes a few days to few weeks to reach a pharmacologically steady state in vivo [31], as we demonstrated in the present study. This was statistically significant (*p* < 0.0001) when compared DMBA only with the concurrent administration of genistein with the painting of carcinogen before the establishment of a well-defined tumor.

The present study reported highly significant differences (*p* < 0.0001) in both DNA ploidy state and SPF value in the HBPs between DMBA-treated group and DMBA + genistein-treated group. This supports the antitumor chemoprotective role of genistein during DMBA-induced carcinogenesis process. Our results are comparable to the concept of using the nuclear morphometric features and DNA ploidy by flow cytometry as prognostic markers of cancer [34, 35]. The principal action of the genistein was to slow down cell transit through S phase, which was observed as the suppression of cell entrance to G2 phase [36]. Normally, DNA damage during S phase is sufficient to slow transit through the S phase or cause a block in G2 to allow the repair of the potentially lethal damage [37]. Moreover, genistein modulates the cell cycle through the progression of the proliferating cells through the S and G2 phases and the transition of cells from the G0 to G1 [38]. This modulation reduces the aneuploidy and the activity of SPF during treatment. It depends, as well, on the tumor cell type and is drug concentration specific. Another study demonstrated that genistein increased the numbers of intermediate and superficial cells and reduced atrophic crowded parabasal cells [39]. Genistein has been demonstrated to modify the activity of key cell proliferation and survival pathways, such as those controlled by the protein kinase B, the signal transducers and activators of the transcription protein, nuclear factor-κB, and cyclooxygenase-2 [40]. In contrast, Le Donne and colleagues found no significant change in SPF value and DI using genistein treatments in comparison with hyaluronic acid [41], however, they applied it vaginally for assessment of atrophic epithelium in postmenopause and not tumors. Furthermore, the aneuploid status was normalized by genistein. Comparable results were obtained that all diploid tumors had S-phase percentage less than those of the aneuploid neoplasms, but with no significant difference [35].

The growth inhibitor action of genistein has been distinguished in several cancer cells by arresting the cell cycle which leads to cessation of cell proliferation. It arrests the cell cycle in both gap 1 (G1) and gap 2/mitosis (G2/M) [7]. The programmed cell death, apoptosis, is induced in several cell lines by genistein [8].

The result of the present study supports the hypothesis that DNA aneuploidy may contribute to the ability of the tumor to change into malignancy after alteration to recurrent tumors and finally to carcinoma [42]. Additionally, abnormal DNA amounts can be detected by the flow cytometric analysis. Therefore, this method could be applied to the study of oral cancer development and the effects of drugs strategies to prevent or decrease the risk of malignant transformation [43, 44].

## Conclusion

Genistein administration could be considered as a chemotherapeutic agent against oral squamous cell carcinoma development, through reduction of activated DNA proliferation activity and carcinogenesis process. Future research is required to prove the possible usage of genistein as a valid chemotherapeutic agent against squamous cell carcinoma in combination with other treatment strategies.

## Data Availability

All data and materials are available upon request.

## Abbreviations

SCC: Squamous cell carcinoma
OSCC: Oral squamous cell carcinoma
DMBA: 7,12-Dimethylbenz[a]anthracene
BM: Buccal mucosa
FCM: Flow cytometry
FMA: Flow cytometry analysis
FACS: Fluorescence-activated cell sorting
SPF: S-phase fragment
G1: Gap 1 phase
G2/M: Gap 2/mitosis phase
G0: GAP 0 phase
2N: Diploid
4N: Tetraploid state
DI: DNA index
SPF: Synthesis phase fraction
HBP: Hamster buccal pouch
PFA: Paraformaldehyde
H&E: Hematoxylin and eosin
SD: Standard deviation
